# NetMet: A Network-Based Tool for Predicting Metabolic Capacities of Microbial Species and their Interactions

**DOI:** 10.3390/microorganisms8060840

**Published:** 2020-06-03

**Authors:** Ofir Tal, Gopinath Selvaraj, Shlomit Medina, Shany Ofaim, Shiri Freilich

**Affiliations:** Institute of Plant Sciences, Newe Ya’ar Research Center, The Agricultural Research Organization, Ramat Yishay 30095, Israel; ofirt@volcani.agri.gov.il (O.T.); gopiselva@gmail.com (G.S.); shmedina@volcani.agri.gov.il (S.M.); shashany@gmail.com (S.O.)

**Keywords:** metabolic networks, microbial interactions, genomics, environment, network modeling, expansion algorithm, simulation

## Abstract

Metabolic conversions allow organisms to produce a set of essential metabolites from the available nutrients in an environment, frequently requiring metabolic exchanges among co-inhabiting organisms. Genomic-based metabolic simulations are being increasingly applied for exploring metabolic capacities, considering different environments and different combinations of microorganisms. NetMet is a web-based tool and a software package for predicting the metabolic performances of microorganisms and their corresponding combinations in user-defined environments. The algorithm takes, as input, lists of (i) species-specific enzymatic reactions (EC numbers), and (ii) relevant metabolic environments. The algorithm generates, as output, lists of (i) compounds that individual species can produce in each given environment, and (ii) compounds that are predicted to be produced through complementary interactions. The tool is demonstrated in two case studies. First, we compared the metabolic capacities of different haplotypes of the obligatory fruit and vegetable pathogen *Candidatus* Liberibacter solanacearum to those of their culturable taxonomic relative *Liberibacter crescens*. Second, we demonstrated the potential production of complementary metabolites by pairwise combinations of co-occurring endosymbionts of the plant phloem-feeding whitefly *Bemisia tabaci*.

## 1. Introduction

The introduction species’ metabolism is adapted to fulfill their nutritional needs in natural environments. That is, in a viable environment, organisms can produce a set of metabolites required for their growth and development from the available nutrients. The metabolic capacities of an individual species depend on its enzymatic profile. The genomics data hoard the information on species’ enzymatic profiles. Computational tools enable the processing of genomics data into a metabolic network representation, and the subsequent simulation of metabolic capacities in selected environments [1]. Given a representation of species’ genome as a metabolic network, computational simulations allow the addressing of the influence of environmental inputs (nutritional resources) on the metabolic capacities of a species. Metabolic capacities can be estimated in terms of growth rate, as well as the ability to produce selected metabolites [1,2]. One of the key algorithms for predicting the production profiles of metabolites by a species is the expansion algorithm, developed by Ebenhöh et al. [3]. The algorithm generates a set of all possible metabolites that can be produced, given a set of starting compounds (representing “environment” or “medium”) and a set of feasible reactions (based on enzymes drawn from genomic data of participating organisms). The expansion algorithm starts with a set of one or more biochemical compounds (environment) acting as source metabolites for a feasible reaction, i.e., a reaction for which all required substrates are available. This reaction is selected out of the reaction pool and added to the network. In an iterative process, the products of the chosen reaction are turned into the new substrates, and so on. Processing of the starting-point compounds by relevant reactions increases the number of available compounds that can act as substrates for other previously inactivated reactions. The network stops expanding when there are no more feasible reactions. The algorithm hence allows one to carry out a comparative analysis of different species’ metabolic capacities [4,5], to predict the growth potential in different niches [6], and to estimate the potential effect of genetic and environmental perturbations [7,8].

In natural habitats, the metabolic capacities of an organism per se are typically not sufficient to meet all their nutritional requirements. Towards fulfilling these requirements, species are frequently involved in metabolic exchanges with other co-inhabiting organisms. The genomics data hoard the information not only on the enzymatic profiles, but also on the corresponding gaps, in terms of missing genes or enzymes in metabolic pathways. Genomics-based approaches are increasingly applied for going beyond the single species and further exploring trophic interactions [9,10,11]. In recent years, the expansion algorithm has been implemented into several tools and applied in several studies for the prediction of interactions between microorganisms, including competitive and cooperative interactions [12,13,14,15,16,17,18]. Examples include the NetCmpt tool [19] the output of which is a score estimating the level of metabolic overlap (competition) between pairs of microorganisms, and the NetCooperate tool [20], the output of which is two different scores estimating the extent of cooperative/supportive interactions. Here, we report on the NetMet tool—a python implementation of the expansion algorithm. NetMet takes as input a predefined list(s) of environmental inputs (natural growth media), and genomics-based lists of species-specific enzymes (used to expand the network of metabolic reactions). For each given species in each given environment, the algorithm generates as output the production profile of the full set of metabolites, also highlighting essential cellular compounds. Essential cellular compounds include molecules that are highly likely to be critical for the growth and/or maintenance of most species, including, for example, nucleic acids and amino acids, and were defined based on compilation of previous studies [6]. In addition, the user can generate predictions for complementary metabolites that are formed through exchange interactions between species combinations. As species interactions are dynamic and, to a large extent, reflect the specific environment, the tool allows for the examining of the complementary profiles in different environments. NetMet is available online at https://freilich-lab-tools.com/.

## 2. Materials and Methods

### 2.1. Description of the Expansion Algorithm and its Implementation in the NetMet Tool

**Figure 1:**
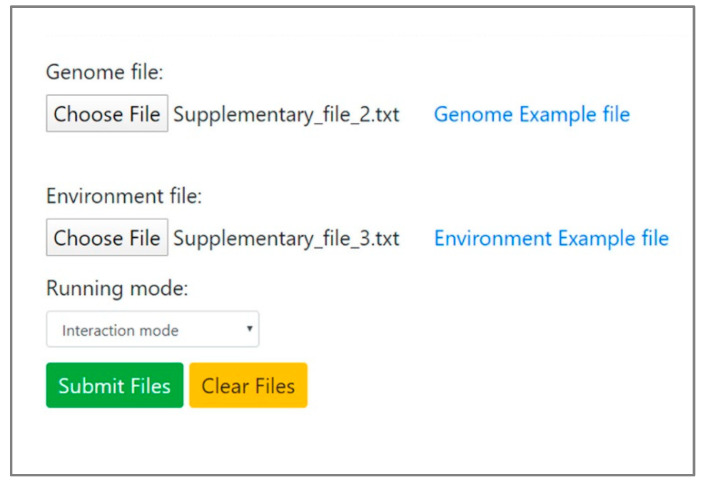
The data input panel of the NetMet web tool interface. The user uploads two network files and selects analysis mode from the drop-down menu.

NetMet receives as input two files: a file representing species’ metabolic capacities, as encoded by their genomes (termed ‘Genomes file’), and another file describing selected metabolic environments (termed ‘Environment file’). The Genome file contains lists of species-specific enzymatic reactions (EC numbers), with each species in a separate line. EC accession identifiers are used in several metabolic databases, including KEGG [21] and MetaCyc [22]. In newly sequenced genomes, assignment into EC accessions is supported by several tools and platforms, including Blast2GO [23], JGI (IMG/M) [24] and GhostKOALA [25]. The Environment file contains a list of compounds represented by KEGG Compound accession [21,26,27], each environment in a separate line. KEGG compound accessions are compatible with additional resources for metabolic data, such as MetaCyc [28]. Input files are space-delimited text files. The Instruction file for the construction of the Genome and Environment files is provided in Supplementary file 1 (also available on the website). Several examples of artificial and natural environments are provided. Input files used for producing the case studies in the Results section are provided in Supplementary files 2–5. Given the input files, the NetMet tool calculates the expanded metabolic networks for all corresponding Genome x Environment combinations, using the implemented expansion algorithm, as previously described [5,17,18]. Enzymatic reactions will be considered to take place if all respective substrates are available. Co-factors are not defined as reactants (substrates/products), with the exception of their own biosynthesis pathways. For example, 2.1.1.13 and 2.1.1.14 are defined as cobalamin-dependent and cobalamin-independent methionine syntheses, respectively [29,30]. A reaction’s occurrence will rely only on the availability of the main reactants (for example, as defined in KEGG: https://www.genome.jp/dbget-bin/www_bget?ec:2.1.1.13/ and https://www.genome.jp/dbget-bin/www_bget?ec:2.1.1.14, under the ‘substrates’ field) and not on the presence of the co-factor (cobalamin). In cases of EC accessions with multiple associated reactions, all reactions are considered. The user defines whether the calculation is carried out in a single-species or interaction mode (Figure 1).

### 2.2. Single-Species Mode

**Figure 2:**
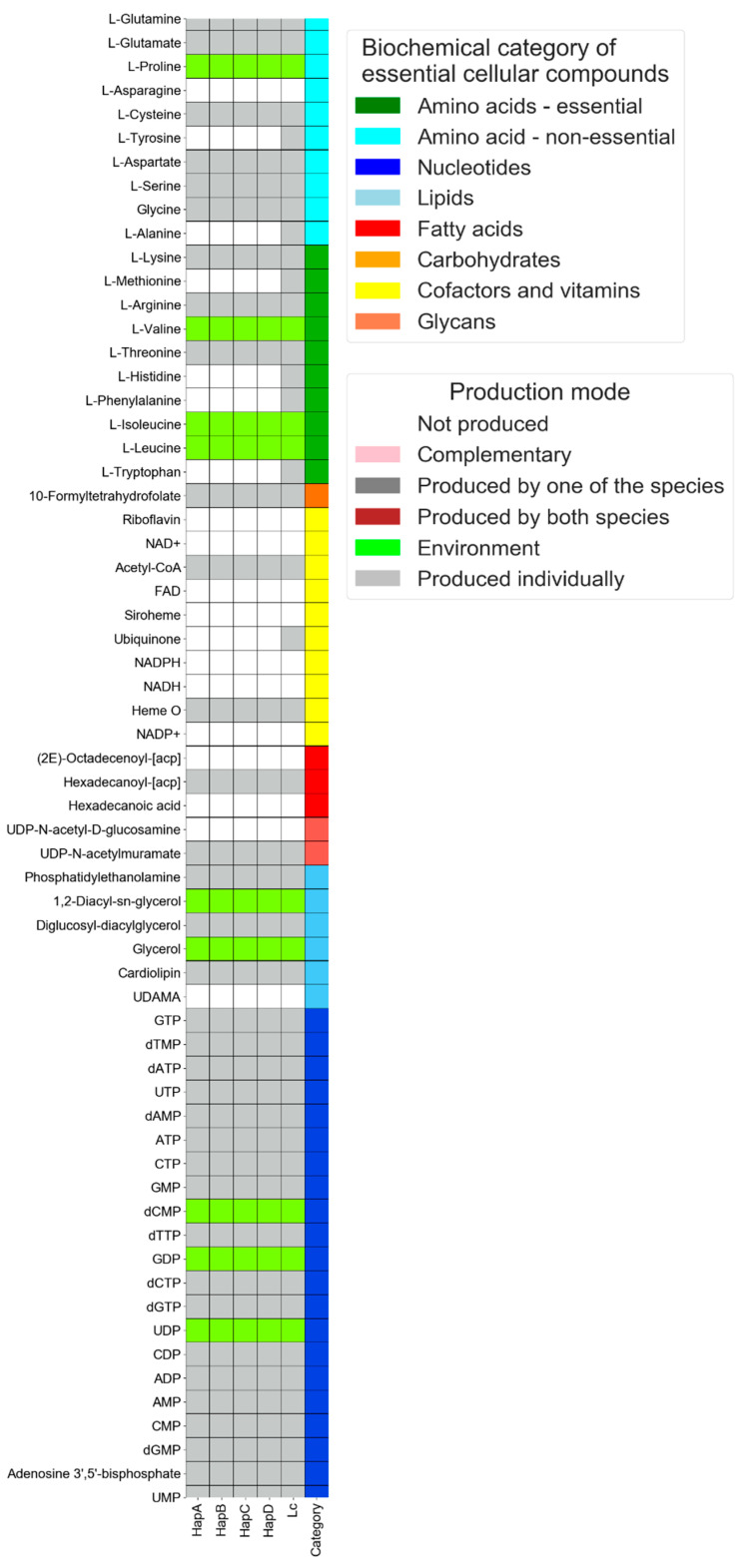
Profiles of the predicted production of cellular building blocks by plant pathogens from the *Liberibacter* genus in a proxy of the natural environment. X-axis—HapA-D: haplotypes A, B, C and D of *Candidatus* Liberibacter solanacearum; LC—*Liberibacter crescens*. Y-axis—key cellular building blocks.

In this mode, the prediction of metabolic capacities is made based on the individual species’ network in the given environment(s). Output files include the corresponding metabolic network for each species in each environment, provided as a list of metabolites represented by their KEGG compound accession. Out of the full list of metabolites, a graphical representation highlights a subset of pre-defined essential cellular compounds (exemplified in Figure 2), including amino acids, nucleic acids and co-factors [18].

### 2.3. Interaction Mode

**Figure 3:**
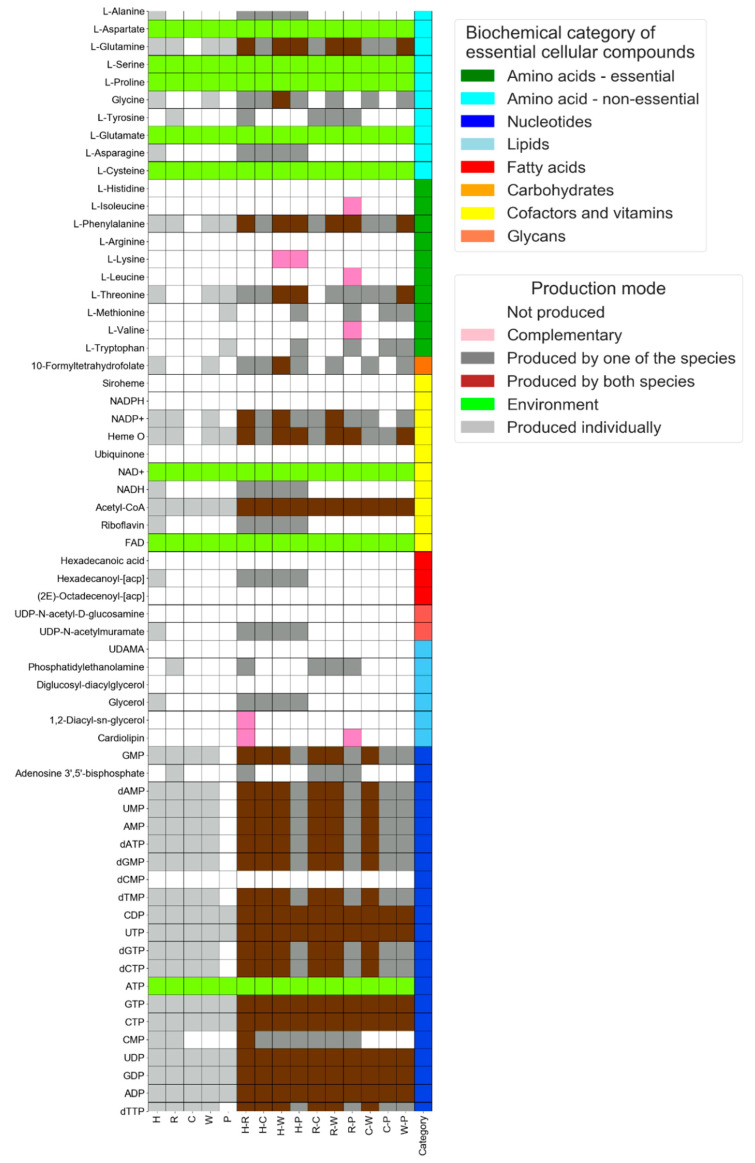
Profiles for the predicted production of cellular building blocks by co-residing symbionts of *Bemisia tabaci* and their respective pairwise combinations. X-axis: H, R, C, W & P—*Hamiltonella*, *Rickettsia*, *Wolbachia*, *Cardinium*, and *Portiera* respectively. Y-axis: key cellular building blocks.

Interaction mode is an extension of the single-species mode, providing a list of metabolites that are produced through exchange reactions between all pairwise combinations of given species. Such metabolites are termed ‘complementary metabolites’, and are defined as those that are formed by species combinations, but not by the corresponding individual species. Co-growth is, practically, the representation of two species as a corresponding merged metabolic network, consisting of all available reactions of each of the collaborating organisms [18]. Simulations are then carried out as in the ‘single-species’ mode: after a reaction is simulated, assuming all its substrates are available in the environment, the resulting and previously lacking products are now added to the compound pool representing the environment. New compounds that are produced in each iteration enable additional reactions to take place. Iterations continue until no new compounds are added to the environment. Complementation is predicted through a three-stage model described in [18]: (1) constructing a combined set of metabolic reactions (EC accessions) for each pairwise combination; (2) simulating co-growth of pairs of species in the given environment(s); and (3) comparing the set of metabolites produced by the combined genomes to those formed by the individual genomes. Output files include the list of metabolites for single-species and species combinations (complementary metabolites), a graphical representation highlighting the complementary metabolites within the subset of pre-defined essential cellular compounds (exemplified in Figure 3), and a log file containing the process sequences with data.

NetMet is available at https://freilich-lab-tools.com/. The package was implemented in Python3.7. Extended information on usage and construction of the input file can be found in Supplementary file 1.

## 3. Results

### 3.1. Single-Species Mode: Comparative Analysis of the Metabolic Capacities of Liberibacter Species That Have Undergone Genomic Reduction

*Candidatus* Liberibacter solanacearum is the suspected causal agent of several plant diseases and is restricted intracellularly to the plant phloem sieve elements [5]. Several haplotypes of the pathogen have been identified, and the genomes of haplotypes A, B, C and D were sequenced [5,31,32,33]. These sequenced genomes are relatively small, at <1.4 MB [5], explained by rapid evolutionary adaptation to an obligatory intracellular lifestyle [34]. Reduced genome size is characteristic of obligatory intracellular symbionts and pathogens, assumed to reflect an adaptation to the well-defined environmental conditions in which these bacteria can multiply [35]. None of these species’ haplotypes were successfully grown in artificial media. Currently, the only culturable *Liberibacter* species is *Liberibacter crescens*, also a fruit pathogen, which possesses a slightly larger genome (1.5 MB) and has a broader metabolic capacity [36]. Here, we demonstrate the use of NetMet for delineating the differences in metabolic performances of the *Ca*. L. solanacearum haplotypes from those of the relatively versatile, closely related species *L. crescens*. Given the sets of the enzymes (as a list of ECs) of the above mentioned two species (four haplotypes of *Ca*. L. solanacearum and *L. crescens*) and a list of environmental inputs representing nutritional resources (Supplementary files 2 and 3, respectively, based on [5]), the NetMet tool allows for the comparing of the metabolic capacities of each strain in the given environment. In the current example, the two input files contain (i) the EC lists of the different haplotypes (A, B, C and D) of *Ca*. L. solanacearum and of *L. crescens*, and (ii) a list of metabolites as its environment [5]. We defined this nutritional environment based on a computational approach: the NetSeed algorithm that predicts metabolic resources based on genomic information [37]. The environment is a proxy for the compilation of nutrients provided by the host psyllid in the environment of *L. crescens* and is composed of 453 compounds (based on [5]).

For each of the haplotypes of *Ca*. L. solanacearum and *L. crescens*, we simulated metabolic activity in the predicted environment. Regarding the output files, the full set of compounds produced for each strain in an excel file and simulation .txt log file are compressed into a zipped folder (Supplementary file 6). Besides this, we listed a sub-set of essential cellular compounds predicted to be produced by individual species (Figure 2). Our analysis shows that, in contrast to *L. crescens*, all *Ca*. L. solanacearum haplotypes have lost their ability to produce the electron carrier ubiquinone, and are also unable to produce the L-amino acids alanine, phenylalanine, tyrosine, tryptophan, methionine and histidine (Figure 2), in accordance with Katsir et al. [5].

### 3.2. Interaction Mode: Delineating Exchange Interactions between Endosymbionts of Phloem-Feeding Whitefly Bemisia tabaci

The phloem-feeding whitefly *Bemisia tabaci* (Hemiptera: Aleyrodidae) harbors an obligatory symbiotic bacterium (*Portiera*), as well as varying combinations of facultative symbionts (*Rickettsia*, *Hamiltonella*, *Cardinium* and *Wolbachia*). The obligatory symbiont *Portiera* fulfills several critical functions, including complementing the production of essential amino acids (for example, [18,38,39,40]). Metabolic interactions among these symbionts can potentially shed light on the functional significance of their varying combinations and observed co-occurrence patterns [41]. Here, this well-defined bacterial community serves as a case study for demonstrating the use of the NetMet tool in the prediction of metabolic interactions among microorganisms. Though the specific case study focuses on interactions among symbionts, not considering the whitefly, the tool can be similarly applied in exploring host–symbiont interactions. The first input file (Supplementary file 4) contains the lists of enzymes (as ECs) from the genome sequences of these five common *B. tabaci* symbionts genera: *Portiera*, *Rickettsia*, *Hamiltonella*, *Cardinium* and *Wolbachia* [18]. All these species were reported to inhabit the whitefly’s bacteriocytes—designated cells forming an organ (bacteriome) in which the insect harbors its symbionts. Simulations were carried out in a “bacteriocyte-like” environment, which includes the set of metabolites in the second input file (Supplementary file 5) that are predicted to be produced by the host [18]. To predict potential complementation patterns between these co-residing symbionts, NetMet carries out co-growth simulations for pairwise combinations formed between the input genomes in the given environment(s). A metabolite is defined as “complementary” if its synthesis requires a combination of the metabolic networks of two input genomes (i.e., cannot be produced by individual members of the combination). The output file for this example simulation is in Supplementary file 7 (the zipped folder, containing lists of compounds for individual and pairwise combinations of these genera in an excel file and a simulation .txt log file). The production of essential cellular compounds by individual and pairwise combinations was illustrated in Figure 3 (also reported in [18]). Complementary metabolites by any pairwise combinations are shown in Figure 3 as pink-colored cells. Lysine is produced by the combination of both *Portiera* and *Hamiltonella*, and *Hamiltonella* and *Wolbachia*. The combination of *Portiera* and *Rickettsia* demonstrates the production of cardiolipin and the amino acids valine, leucine and isoleucine. Likewise, the combination of *Hamiltonella* and *Rickettsia* demonstrates the production of 1,2-Diacyl-sn-glycerol and cardiolipin.

## 4. Discussion

Network analysis has become an essential component in the study of microbiology, and a complementary tool for the interpretation of genomic data. Here we demonstrate the use of NetMet in carrying out two types of such metabolic network analysis, under the single-species and interaction modes: the single species mode (Figure 2) demonstrates the use of the tool for generating genomic-based predictions of species’ metabolic capacities in a defined environment; the interaction mode (Figure 3) demonstrates the use of the tool for generating genomic-based predictions of metabolic interactions by potentially naturally occurring combinations of species in a particular environment. NetMet can assess these potentials in multiple environments simultaneously (as shown in the example files in the website).

The NetMet tool relies on the description of species’ enzymatic sets. Since metabolic capacities are based solely on genomic data and the topological structure of the respective metabolic networks, it provides qualitative, binary predictions for the production or absence of a metabolite [17,42,43]. Alternative quantitative approaches for metabolic modeling, mainly those that are based on Constraint-Based Modeling (CBM), produce quantitative estimates for the metabolite consumption/production rate. Though CBM is being increasingly applied in the study of interactions in natural ecosystems [1,11,38,44,45], the computational complexity associated with such quantitative approaches, and the partiality of available data in most studies concerning natural ecosystems (for example, regarding the level of gene expression and/or metabolite accumulation), introduces many assumption-based parameters. The topological-based qualitative approaches, as applied here, thus provide a powerful yet relatively straightforward framework for the analysis of genome-wide ‘omics’ data [17,42,43].

Here, we present NetMet, a web-based tool for easily producing predictions of metabolic capacities and exchanges between microorganisms present in a particular environment. Simulations take into account specific environments, hence reflecting the common notion that interactions are dynamic and can vary with the addition or depletion of nutrients [18,46]. Model-derived predictions should be treated as educated ‘leads’ that are useful for the formulation of testable hypotheses. Such predictions allow researchers to delineate biological signals and ecological relevance from complex data.

## 5. Conclusions

The NetMet tool allows a systematic view of microbial function and interactions, leading to the prediction of metabolic performances and complementary interactions, hence providing a tool for generating testable hypotheses of metabolic interactions in bacterial communities in predefined environments. In the purview of microbial ecology, NetMet allows the interpretation of already existing genomics data, and deciphers associations between microbial communities and their natural environments.

## Supplementary Materials

The following are available online at https://www.mdpi.com/2076-2607/8/6/840/s1, Supplementary file 1. Docx file containing instructions for the usage of NetMet tool in the website. Supplementary file 2. Lists of ECs from genomic data of four haplotypes of *Ca*. L. solanacearum and *L. crescens* (Results). Supplementary file 3. List of environmental compounds of four haplotypes of *Ca*. L. solanacearum and *L. crescens* as KEGG compound numbers (Results). Supplementary file 4. Lists of ECs from genomic data of different symbionts of *Bemisia tabaci* (Results). Supplementary file 5. List of environmental compounds of symbionts of *Bemisia tabaci* as KEGG compound numbers (Results). Supplementary file 6. Results from ‘single-species mode’ of the web-based tool NetMet using Supplementary file 2 and 3 as inputs. Supplementary file 7. Results from ‘interaction’ mode of the web-based tool NetMet using Supplementary file 4 and 5 as inputs.
