# Supplementary material for: NetMet: A Network-Based Tool for Predicting Metabolic Capacities of Microbial Species and their Interactions
Source: Microorganisms. 2020 Jun 3;8(6):840. doi: 10.3390/microorganisms8060840 (PMC7356744; doi:10.3390/microorganisms8060840)
Supplement: Supplementary file 1 [file microorganisms-08-00840-s001.zip › Supplementary_files/Supplementary_file_1.docx]

**Instructions File:**

**Genome files** include species-specific enzymatic reaction content in their EC format. Each species in a separate line. First string is species' id followed by EC accessions that are space-delimited.

How do I get the EC content list? For non-sequenced species, the EC content can be constructed according to BLAST results and annotation tools such as BLAST2GO.
For sequenced species, the EC content can be retrieved from sites such as the DOE JGI site ([genome.jgi.doe.gov](http://genome.jgi.doe.gov/)). Following choosing a specific species (e.g.,
<https://img.jgi.doe.gov/cgi-bin/m/main.cgi?section=TaxonDetail&page=taxonDetail&taxon_oid=645951868> )
look at enzymes (under genome statistics). This leads to species-specific lists of EC numbers (<https://img.jgi.doe.gov/cgi-bin/m/main.cgi?section=TaxonDetail&page=enzymes&taxon_oid=645951868#enzyme=results%3D298%26startIndex%3D0%26sort%3DEnzymeID%26dir%3Dasc%26c%3DEnzymeID%26f%3D%26t%3Dtext>)

**Environment files** include lists of metabolites in their KEGG accessions. Each environment in a separate line. First string is environment's id followed by metabolite accessions that are space-delimited. An example file is provided in the website including a minimal environment with carbon and nitrogen sources (glucose and ammonium; Env_1 in the example file in the website), carbon source only (Env_2), nitrogen source only (Env_3), and a rich environment with multiple carbons, co factors, amino acids and additional compounds (Env_4). In addition, below we provide examples for four representations of environments including artificial defined growth media (M9; ATCC medium 2511 [1]) and natural compiled based on experimental analyses (Tomato_Phloem_Env; [2,3,4,5,6]) or computational based proxy (Cucumber_Root_Env, Bulk_Soil_Env; [7]).

M9 C13558 D02050 C13563 C12538 C00031 C12505 C08130 C00378

Tomato_Phloem_Env C00049 C00025 C00152 C00065 C00064 C00037 C00188 C00041 C00334 C00183 C00407 C00079 C00123 C00047 C00089 C00031 C10906 C00238 C00009 C00698 C00076 C00305 C01330 C00023 C00038 C00034 C00070

Cucumber_Root_Env C05375 C18678 C05212 C11971 G00502 G00089 C02920 C00371 G00036 C05847 C02949 C00525 C00550 C12210 C00643 C06790 C08539 G00102 C01866 C07027 C05465 C16353 C05682 C05475 C07480 C05905 C10443 G00077 C00410 C11088 C01609 C12135 C01322 C05629 C00854 G00031 C20138 C05469 C02035 C02380 C01416 C12414 C05122 C03664 C12405 C00996 G00066 C00515 C00739 C05477 C06202 C12425 G10770 C01110 C01880 C00328 C11909 C03806 C00250 C16641 C16226 C00978 C05604 C04706 C06556 C05473 C00770 C05280 C15605 C15699 C01062 C06635 C03360 C15673 C03239 C02378 G00103 C01112 C05279 G00068 C11955 C05453 C10419 C11921 C11476 C11963 C05471 C14103 G10584 C03772 G00097 C19879 C16396 C01189 G00081 C04598 C15785 C11942 C06030 C00314 C06672 C05539 C00729 G00054 C11979 C07054 C05466 C18680 C05651 G00072 C18032 C05451 C02029 C01921 C16146 C02729 C02780 C02656 C12650 C05479 C07073 C00488 C00338 C17268 G00084 C11863 C00414 C07645

Bulk_Soil_Env C11519 C16361 C04573 C00777 C01888 C00725 C07114 C00643 C20333 C09822 C06719 C05984 C02371 C16567 C15986 C02364 C16014 C02359 C05578 C00353 C02380 C01548 C06584 C00515 C17439 C01880 C00916 C00250 C06670 C05427 C16267 C00479 C02362 C00606 C02357 G10546 C02050 C12449 C06104 C19929 C16396 C02575 C02880 C00314 C17440 C00300 G01318 C00305 C00861 C06588 C06536 C00534 C05651 C00486 C01708 C02090 C09243 C06899 C01438 C07648 C00969 C01147 C20253 C00940 C00414 C04178 C07645

References:

1. American Type Culture Collection medium 2511: https://www.atcc.org/~/media/B3590BBD1DF74203B0E8F0890ECF974F.ashx
2. Valle EM, Boggio SB, Heldt HW. Free amino acid composition of phloem sap and growing fruit of *Lycopersicon esculentum*. Plant Cell Physiol. **1998**, 39:458-461
3. Luis G, Hernández C, Rubio C, González-Weller D, Gutiérrez Á, Revert C, Hardisson A. Trace elements and toxic metals in intensively produced tomatoes (*Lycopersicon esculentum*). Nutr Hosp. **2012,** 27:1605-1609. doi: 10.3305/nh.2012.27.5.5944. PMID: 23478712.
4. Osorio S.; Ruan Y.-L.; Fernie A.R. An update on source-to-sink carbon partitioning in tomato. Front Plant Sci 2014, 5, 516-516, doi:10.3389/fpls.**2014**.00516
5. Alfocea, F.P.; Balibrea, M.E.; Alarcón, J.J.; Bolarín, M.C. Composition of Xylem and Phloem Exudates in Relation to the Salt-tolerance of Domestic and Wild Tomato Species. J. Plant Physiol. **2000**, 156, 367-374, doi:https://doi.org/10.1016/S0176-1617(00)80075-9
6. Wolterbeek, H.T.; Willemse, P.C.M.; Van Die, J. Phloem and xylem import of water and solutes in tomato fruits. *Acta Bot. Neerl.* **1987**, *36*, 295-306, doi:10.1111/j.1438-8677.1987.tb02008.x
7. Ofaim S, Ofek-Lalzar M, Sela N, Jinag J, Kashi Y, Minz D, et al. Analysis of microbial functions in the rhizosphere using a metabolic-network based framework for metagenomics interpretation. Front. Microbiol. **2017**,8:1606
